# Poly[[di­aqua­(μ_4_-benzene-1,2,4,5-tetra­carboxyl­ato)tetrakis­(1*H*-imidazole-κ*N* ^3^)dicopper(II)] *N*,*N*-di­methyl­formamide monosolvate]

**DOI:** 10.1107/S1600536813010246

**Published:** 2013-04-20

**Authors:** Xiao-Fei Zhou, Hong-Ping Xiao, Ya-Juan Zhao, Xin-Hua Li

**Affiliations:** a College of Chemistry and Materials Engineering, Wenzhou University, Wenzhou, Zhejiang 325035, People’s Republic of China

## Abstract

The asymmetric unit of the polymeric title compound, {[Cu_2_(C_10_H_2_O_8_)(C_3_H_4_N_2_)_4_(H_2_O)_2_]·C_3_H_7_NO}_*n*_, contains two independent Cu^II^ ions, each coordinated by one water mol­ecule, two imidazole N atoms and two carboxyl­ate O atoms from benzene-1,2,4,5-tetra­carboxyl­ate anions in a distorted square-pyramidal geometry. The benzene-1,2,4,5-tetra­carboxyl­ate anion bridges four Cu^II^ ions, forming a polymeric sheet parallel to (010). In the crystal, extensive N—H⋯O and O—H⋯O hydrogen bonds link the polymeric sheets and di­methyl­formamide solvent mol­ecules into a three-dimensional supra­molecular structure.

## Related literature

For background to the benzene-1,2,4,5-tera­carboxyl­ate ligand in coordination polymers, see: Andruh *et al.* (2011 ▶); Clarke *et al.* (2012 ▶); Jiang *et al.* (2008 ▶); Aghabozorg *et al.* (2007 ▶); Chu *et al.* (2001 ▶); Liu & Ding (2007 ▶); Wu *et al.* (2006 ▶). For related structures, see: Zhan & Li (2010 ▶); Luo *et al.* (2007 ▶); Yang *et al.* (2004 ▶). For the synthesis, see: Zhao *et al.* (2010 ▶).
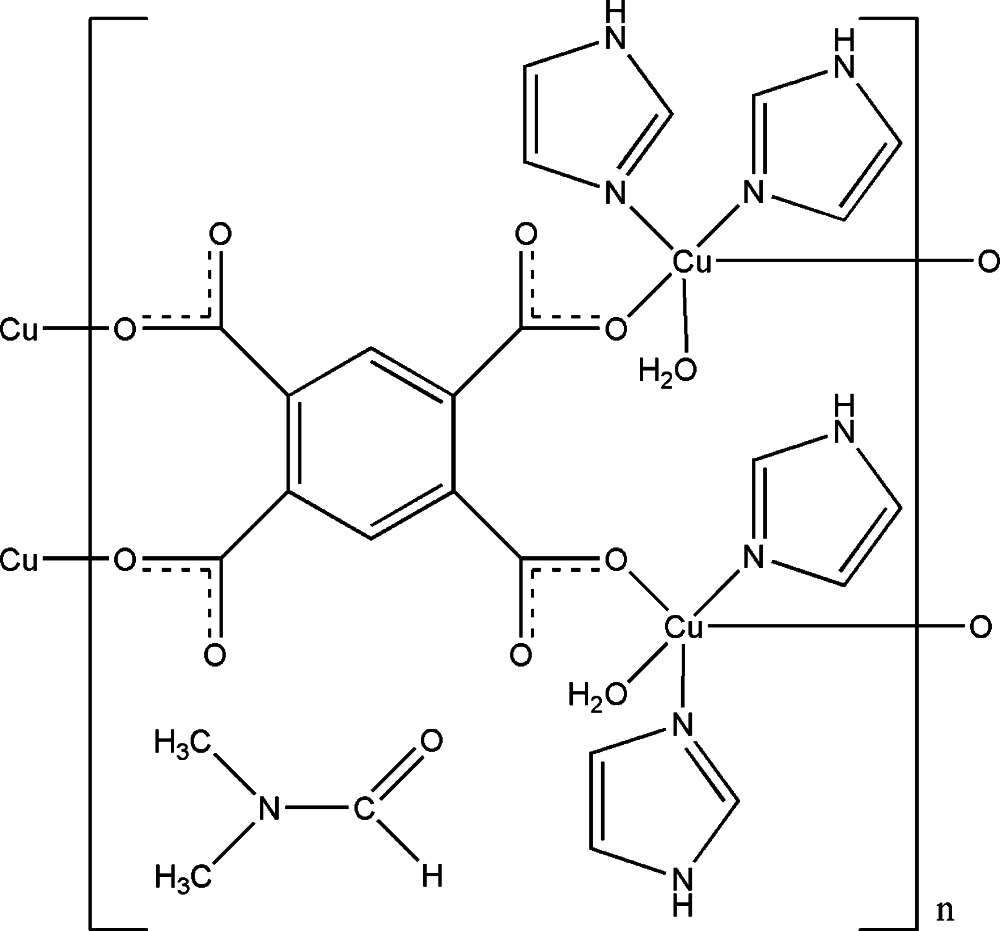


## Experimental

### 

#### Crystal data

[Cu_2_(C_10_H_2_O_8_)(C_3_H_4_N_2_)_4_(H_2_O)_2_]·C_3_H_7_NO
*M* *_r_* = 758.65
Monoclinic, 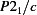

*a* = 8.999 (4) Å
*b* = 19.296 (8) Å
*c* = 18.926 (7) Å
β = 110.590 (18)°
*V* = 3076 (2) Å^3^
*Z* = 4
Mo *K*α radiation
μ = 1.46 mm^−1^
*T* = 298 K
0.23 × 0.21 × 0.15 mm

#### Data collection

Bruker APEXII area-detector diffractometer
Absorption correction: multi-scan (*SADABS*; Bruker, 2001 ▶) *T*_min_ = 0.731, *T*_max_ = 0.811
17289 measured reflections
5445 independent reflections
4149 reflections with *I* > 2σ(*I*)
*R*_int_ = 0.038

#### Refinement

*R*[*F* ^2^ > 2σ(*F* ^2^)] = 0.040
*wR*(*F* ^2^) = 0.153
*S* = 1.11
5445 reflections
426 parameters
H-atom parameters constrained
Δρ_max_ = 0.51 e Å^−3^
Δρ_min_ = −0.60 e Å^−3^

### 

Data collection: *APEX2* (Bruker, 2007 ▶); cell refinement: *SAINT* (Bruker, 2007 ▶); data reduction: *SAINT*; program(s) used to solve structure: *SHELXTL* (Sheldrick, 2008 ▶); program(s) used to refine structure: *SHELXTL*; molecular graphics: *SHELXTL*; software used to prepare material for publication: *SHELXTL*.

## Supplementary Material

Crystal structure: contains datablock(s) I, global. DOI: 10.1107/S1600536813010246/xu5689sup1.cif

Structure factors: contains datablock(s) I. DOI: 10.1107/S1600536813010246/xu5689Isup2.hkl

Additional supplementary materials:  crystallographic information; 3D view; checkCIF report

## Figures and Tables

**Table 1:** Selected bond lengths (Å)

Cu1—N1	2.003 (4)
Cu1—N3	2.025 (4)
Cu1—O1	1.984 (3)
Cu1—O5^i^	1.985 (3)
Cu1—O9	2.344 (3)
Cu2—N5	1.985 (3)
Cu2—N7	1.980 (3)
Cu2—O3	2.243 (3)
Cu2—O7^ii^	2.003 (3)
Cu2—O10	2.043 (3)

Symmetry codes: (i) 
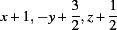
; (ii) 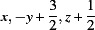.

**Table 2:** Hydrogen-bond geometry (Å, °)

*D*—H⋯*A*	*D*—H	H⋯*A*	*D*⋯*A*	*D*—H⋯*A*
N2—H2*N*⋯O3^iii^	0.86	2.01	2.807 (5)	153
N4—H4*N*⋯O7^ii^	0.86	2.35	3.099 (5)	146
N6—H6*N*⋯O6^i^	0.86	2.03	2.733 (5)	138
N8—H8*N*⋯O2^iv^	0.86	2.35	2.952 (5)	127
N8—H8*N*⋯O4^iv^	0.86	2.26	3.033 (6)	149
O9—H9*B*⋯O11^v^	0.85	2.06	2.909 (5)	177
O9—H9*C*⋯O11^iv^	0.85	2.02	2.805 (6)	153
O10—H10*A*⋯O8^vi^	0.85	1.90	2.655 (4)	147
O10—H10*B*⋯O4	0.85	2.24	2.703 (5)	114

Symmetry codes: (i) 
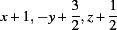
; (ii) 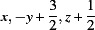; (iii) 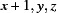; (iv) 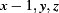; (v) 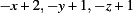; (vi) 
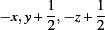
.

**Fig. 1.**
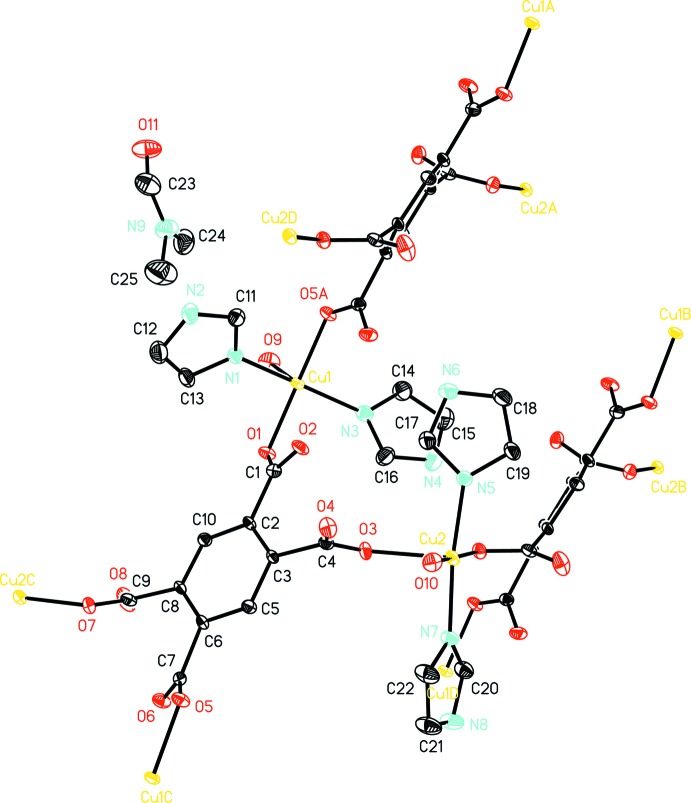
Structure of the (I) with the atom numbering, showing displacement ellipsoids at the 30% probability level. H atoms and DMF molecule have been omitted for clarity.

**Fig. 2.**
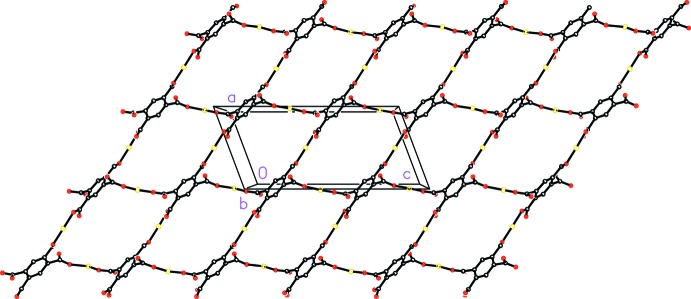
Perspective view of the 2-D sheet of (I).

## supplementary crystallographic information

### Comment

In recent years, many successful implementation of crystal engineering concepts has produced a great deal of coordination and supramolecular networks (Andruh *et al.*, 2011), many of which exhibit unusual and fascinating architectures (Clarke *et al.*, 2012). The benzene-1,2,4,5-teracarboxylate ligand as a multi-connecting ligand is also an excellent candidate for the structuring of coordination networks (Jiang *et al.*, 2008), and comparatively few examples have been reported in relation to applying it to the building of coordination polymers (Aghabozorg *et al.*, 2007; Chu *et al.*, 2001; Liu & Ding, 2007; Wu *et al.*, 2006). Here, the title complex, {[Cu_2_(idz)_4_(btc)(H_2_O)_2_]^.^DMF}_n_ (idz=imidazole, DMF=*N*,*N*-dimethylformamide, btc=benzene-1,2,4,5-tetracarboxylato), (I), represents as a novel example.

As shown in Figure 1, the asymmetric unit of (I) consists of two crystallographically independent Cu^II^ atoms. Both are five-coordinate in a slight distorted tetragonal pyramid geometry, and chemical environment of them are similar. Each Cu^II^ cation is surrounded by two O atoms from two btc tetraanions, two N atoms from two monodentate idz ligands, and one water molecule (O9 or O10) which occupies the axial position. All the bond lengths fall within the typical range of Cu—N bond and Cu—O bond lengths (Luo *et al.*, 2007; Yang *et al.*, 2004).

Cu1 and Cu2 are bridged by a btc tetraanion, which acts as µ_4_-bridge, forming a two-dimensional sheet along the a,*c* axis, as shown in Figure 2. The structure can be regarded as a grid sheets with (4,4) net topology which is constructed through Cu^II^ centers bridged by btc tetraanions. Adjacent two-dimensional sheets are parallel and are linked *via* hydrogen-bonding interactions (see Table 1) through uncoordinated DMF solvent molecules to form a sandwich structure(Zhan & Li, 2010).

### Experimental

A dimethylformamide (DMF) solution (20 ml) of benzene-1,2,4,5-tetracarboxylic acid (0.1 mmol,0.0254 g) was added dropwise to an aqueous solution (10 ml) containing copper sulfate pentahydrate (0.2 mmol,0.0498 g), *N*,*N*-carbonyldiimidazole (0.3 mmol,0.0486 g) and NaOH (0.01 mmol, 0.0004 g) at room temperature (Zhao *et al.*, 2010). The reaction mixture was filtered and the filtrate was left to stand for about three weeks until blue single crystals were obtained (yield 31%, based on Cu).

### Refinement

The nitrogen H atoms were refined subject to the restraint N—H = 0.86 Å. The water H atoms were refined subject to the restraint O—H = 0.85 Å. The other H atoms were positioned geometrically and allowed to ride on their parent atoms at distances of C—H = 0.93-0.96 Å. *U*_iso_(H) = 1.2*U*_eq_(C,N) and 1.5U_eq_(O).

### Crystal data

**Table:**

[Cu_2_(C_10_H_2_O_8_)(C_3_H_4_N_2_)_4_(H_2_O)_2_]·C_3_H_7_NO	*F*(000) = 1552
*M**_r_* = 758.65	*D*_x_ = 1.638 Mg m^−^^3^
Monoclinic, *P*2_1_/*c*	Mo *K*α radiation, λ = 0.71073 Å
Hall symbol: -P 2ybc	Cell parameters from 4538 reflections
*a* = 8.999 (4) Å	θ = 2.3–25.1°
*b* = 19.296 (8) Å	µ = 1.46 mm^−^^1^
*c* = 18.926 (7) Å	*T* = 298 K
β = 110.590 (18)°	Block, blue
*V* = 3076 (2) Å^3^	0.23 × 0.21 × 0.15 mm
*Z* = 4	

### Data collection

**Table:**

Bruker APEXII area-detector diffractometer	5445 independent reflections
Radiation source: fine-focus sealed tube	4149 reflections with *I* > 2σ(*I*)
Graphite monochromator	*R*_int_ = 0.038
φ and ω scans	θ_max_ = 25.1°, θ_min_ = 1.6°
Absorption correction: multi-scan (*SADABS*; Bruker, 2001)	*h* = −10→7
*T*_min_ = 0.731, *T*_max_ = 0.811	*k* = −11→23
17289 measured reflections	*l* = −22→22

### Refinement

**Table:**

Refinement on *F*^2^	Primary atom site location: structure-invariant direct methods
Least-squares matrix: full	Secondary atom site location: difference Fourier map
*R*[*F*^2^ > 2σ(*F*^2^)] = 0.040	Hydrogen site location: inferred from neighbouring sites
*wR*(*F*^2^) = 0.153	H-atom parameters constrained
*S* = 1.11	*w* = 1/[σ^2^(*F*_o_^2^) + (0.094*P*)^2^ + 0.3347*P*] where *P* = (*F*_o_^2^ + 2*F*_c_^2^)/3
5445 reflections	(Δ/σ)_max_ = 0.001
426 parameters	Δρ_max_ = 0.51 e Å^−^^3^
0 restraints	Δρ_min_ = −0.60 e Å^−^^3^

### Special details

**Table:**

Geometry. All e.s.d.'s (except the e.s.d. in the dihedral angle between two l.s. planes) are estimated using the full covariance matrix. The cell e.s.d.'s are taken into account individually in the estimation of e.s.d.'s in distances, angles and torsion angles; correlations between e.s.d.'s in cell parameters are only used when they are defined by crystal symmetry. An approximate (isotropic) treatment of cell e.s.d.'s is used for estimating e.s.d.'s involving l.s. planes.
Refinement. Refinement of *F*^2^ against ALL reflections. The weighted *R*-factor *wR* and goodness of fit *S* are based on *F*^2^, conventional *R*-factors *R* are based on *F*, with *F* set to zero for negative *F*^2^. The threshold expression of *F*^2^ > σ(*F*^2^) is used only for calculating *R*-factors(gt) *etc*. and is not relevant to the choice of reflections for refinement. *R*-factors based on *F*^2^ are statistically about twice as large as those based on *F*, and *R*- factors based on ALL data will be even larger.

### Fractional atomic coordinates and isotropic or equivalent isotropic displacement parameters (Å^2^)

**Table:**

	*x*	*y*	*z*	*U*_iso_*/*U*_eq_	
Cu1	0.49949 (5)	0.66350 (3)	0.40320 (2)	0.02190 (17)	
Cu2	−0.01800 (5)	0.90670 (3)	0.40567 (2)	0.02186 (17)	
O3	0.0200 (3)	0.83286 (14)	0.32205 (15)	0.0254 (6)	
O4	0.1425 (4)	0.90382 (15)	0.26628 (17)	0.0308 (7)	
O2	0.3393 (3)	0.77371 (15)	0.32593 (16)	0.0302 (7)	
O1	0.3008 (3)	0.65963 (14)	0.31383 (15)	0.0265 (7)	
O5	−0.3003 (3)	0.82712 (14)	−0.00888 (15)	0.0259 (7)	
O6	−0.3577 (3)	0.71481 (15)	−0.01062 (16)	0.0289 (7)	
O8	−0.0973 (4)	0.58140 (16)	0.04247 (18)	0.0432 (9)	
O7	−0.0517 (3)	0.66614 (14)	−0.02611 (14)	0.0245 (6)	
O10	0.0235 (3)	0.98875 (15)	0.34694 (16)	0.0313 (7)	
H10A	0.0156	1.0262	0.3690	0.047*	
H10B	0.1166	0.9855	0.3454	0.047*	
O9	0.5490 (4)	0.54417 (17)	0.41879 (19)	0.0445 (8)	
H9B	0.5993	0.5356	0.4651	0.067*	
H9C	0.4612	0.5225	0.4044	0.067*	
N1	0.6196 (4)	0.67888 (18)	0.33332 (19)	0.0276 (8)	
N7	−0.2465 (4)	0.91606 (18)	0.34494 (19)	0.0273 (8)	
N3	0.3720 (4)	0.67091 (19)	0.4723 (2)	0.0316 (9)	
N5	0.2083 (4)	0.91014 (18)	0.47251 (19)	0.0265 (8)	
C7	−0.2741 (4)	0.7665 (2)	0.0167 (2)	0.0227 (9)	
C2	0.1268 (4)	0.7328 (2)	0.2199 (2)	0.0193 (8)	
C4	0.0744 (4)	0.8489 (2)	0.2715 (2)	0.0224 (9)	
C1	0.2665 (4)	0.7223 (2)	0.2915 (2)	0.0247 (9)	
C10	0.0832 (4)	0.6832 (2)	0.1639 (2)	0.0220 (9)	
H10	0.1380	0.6414	0.1718	0.026*	
N8	−0.5031 (4)	0.8996 (2)	0.2980 (2)	0.0443 (11)	
H8N	−0.5959	0.8840	0.2928	0.053*	
C3	0.0416 (4)	0.7953 (2)	0.20941 (19)	0.0173 (8)	
C6	−0.1278 (4)	0.7559 (2)	0.0861 (2)	0.0191 (8)	
C8	−0.0412 (4)	0.6942 (2)	0.09592 (19)	0.0176 (8)	
C5	−0.0858 (4)	0.8058 (2)	0.1428 (2)	0.0220 (9)	
H5	−0.1438	0.8467	0.1360	0.026*	
C13	0.5758 (6)	0.6644 (3)	0.2580 (3)	0.0410 (12)	
H13	0.4916	0.6360	0.2308	0.049*	
C17	0.3268 (5)	0.8762 (2)	0.4620 (3)	0.0358 (11)	
H17	0.3161	0.8477	0.4209	0.043*	
C11	0.7426 (5)	0.7202 (2)	0.3478 (3)	0.0310 (10)	
H11	0.7981	0.7385	0.3953	0.037*	
N6	0.4627 (4)	0.8883 (2)	0.5180 (2)	0.0450 (11)	
H6N	0.5540	0.8715	0.5222	0.054*	
C20	−0.3691 (5)	0.8842 (2)	0.3536 (2)	0.0336 (11)	
H20	−0.3622	0.8547	0.3935	0.040*	
C9	−0.0702 (4)	0.6424 (2)	0.0330 (2)	0.0239 (9)	
N2	0.7785 (5)	0.7327 (2)	0.2862 (2)	0.0396 (10)	
H2N	0.8543	0.7584	0.2834	0.047*	
C14	0.4166 (6)	0.6577 (3)	0.5468 (3)	0.0455 (13)	
H14	0.5065	0.6326	0.5745	0.055*	
C19	0.2747 (5)	0.9448 (3)	0.5398 (3)	0.0404 (12)	
H19	0.2200	0.9727	0.5626	0.048*	
C22	−0.3115 (6)	0.9542 (3)	0.2796 (3)	0.0466 (14)	
H22	−0.2548	0.9828	0.2586	0.056*	
C21	−0.4691 (6)	0.9439 (3)	0.2511 (3)	0.0531 (15)	
H21	−0.5405	0.9635	0.2074	0.064*	
C18	0.4314 (6)	0.9319 (3)	0.5673 (3)	0.0496 (14)	
H18	0.5046	0.9495	0.6116	0.060*	
N4	0.2023 (5)	0.7178 (3)	0.5191 (3)	0.0549 (12)	
H4N	0.1222	0.7405	0.5218	0.066*	
C16	0.2400 (5)	0.7077 (3)	0.4577 (3)	0.0441 (12)	
H16	0.1806	0.7246	0.4101	0.053*	
C15	0.3138 (7)	0.6855 (4)	0.5755 (4)	0.0633 (17)	
H15	0.3192	0.6828	0.6254	0.076*	
C12	0.6730 (6)	0.6973 (3)	0.2298 (3)	0.0525 (14)	
H12	0.6683	0.6960	0.1799	0.063*	
O11	1.2716 (4)	0.4804 (2)	0.4224 (2)	0.0636 (12)	
N9	1.0139 (5)	0.5105 (2)	0.3827 (3)	0.0555 (12)	
C24	0.9597 (8)	0.4537 (4)	0.4157 (4)	0.073 (2)	
H24A	0.9436	0.4139	0.3834	0.110*	
H24B	0.8614	0.4660	0.4217	0.110*	
H24C	1.0376	0.4431	0.4641	0.110*	
C23	1.1617 (7)	0.5176 (3)	0.3867 (3)	0.0634 (17)	
H23	1.1843	0.5542	0.3600	0.076*	
C25	0.8863 (10)	0.5547 (4)	0.3310 (6)	0.132 (4)	
H25A	0.9320	0.5877	0.3066	0.198*	
H25B	0.8327	0.5788	0.3595	0.198*	
H25C	0.8117	0.5261	0.2936	0.198*	

### Atomic displacement parameters (Å^2^)

**Table:**

	*U*^11^	*U*^22^	*U*^33^	*U*^12^	*U*^13^	*U*^23^
Cu1	0.0181 (3)	0.0249 (3)	0.0169 (3)	−0.00076 (19)	−0.00107 (19)	0.0007 (2)
Cu2	0.0243 (3)	0.0221 (3)	0.0170 (3)	0.00021 (19)	0.0045 (2)	0.00088 (19)
O3	0.0329 (15)	0.0251 (17)	0.0207 (14)	−0.0055 (12)	0.0123 (12)	−0.0071 (12)
O4	0.0410 (17)	0.0231 (18)	0.0303 (16)	−0.0099 (13)	0.0149 (13)	−0.0024 (13)
O2	0.0269 (15)	0.0292 (18)	0.0246 (15)	−0.0017 (13)	−0.0034 (12)	−0.0083 (13)
O1	0.0223 (13)	0.0270 (18)	0.0223 (15)	0.0006 (12)	−0.0017 (11)	0.0024 (13)
O5	0.0264 (14)	0.0209 (17)	0.0222 (15)	0.0019 (12)	−0.0017 (11)	0.0024 (12)
O6	0.0229 (14)	0.0276 (18)	0.0291 (16)	−0.0052 (12)	0.0003 (12)	−0.0042 (13)
O8	0.074 (2)	0.0191 (19)	0.0305 (18)	−0.0097 (16)	0.0105 (16)	−0.0003 (14)
O7	0.0304 (14)	0.0242 (17)	0.0184 (14)	−0.0015 (12)	0.0081 (11)	−0.0029 (12)
O10	0.0335 (15)	0.0196 (17)	0.0401 (17)	0.0000 (13)	0.0120 (13)	−0.0002 (13)
O9	0.0472 (19)	0.030 (2)	0.053 (2)	−0.0015 (15)	0.0126 (16)	0.0056 (16)
N1	0.0276 (18)	0.026 (2)	0.0263 (19)	−0.0016 (15)	0.0055 (14)	−0.0036 (15)
N7	0.0266 (18)	0.029 (2)	0.0240 (18)	−0.0003 (15)	0.0060 (14)	0.0036 (15)
N3	0.0258 (18)	0.035 (2)	0.032 (2)	−0.0033 (15)	0.0071 (15)	0.0034 (17)
N5	0.0288 (18)	0.023 (2)	0.0249 (18)	0.0021 (15)	0.0064 (14)	0.0010 (15)
C7	0.0177 (18)	0.029 (3)	0.020 (2)	0.0013 (17)	0.0060 (15)	−0.0044 (18)
C2	0.0225 (18)	0.022 (2)	0.0096 (17)	−0.0001 (16)	0.0008 (14)	0.0012 (15)
C4	0.0200 (18)	0.020 (2)	0.022 (2)	0.0035 (16)	0.0009 (15)	−0.0021 (17)
C1	0.0188 (18)	0.029 (3)	0.024 (2)	0.0015 (17)	0.0045 (16)	−0.0004 (19)
C10	0.0226 (19)	0.019 (2)	0.023 (2)	0.0034 (16)	0.0061 (15)	−0.0001 (17)
N8	0.0246 (19)	0.055 (3)	0.046 (2)	−0.0078 (18)	0.0035 (17)	0.006 (2)
C3	0.0184 (17)	0.019 (2)	0.0125 (18)	−0.0038 (15)	0.0034 (14)	−0.0031 (15)
C6	0.0233 (18)	0.020 (2)	0.0109 (17)	−0.0009 (16)	0.0029 (14)	−0.0005 (15)
C8	0.0197 (17)	0.021 (2)	0.0102 (17)	−0.0042 (16)	0.0028 (13)	−0.0008 (15)
C5	0.0210 (18)	0.021 (2)	0.022 (2)	0.0009 (16)	0.0042 (15)	0.0005 (17)
C13	0.041 (3)	0.057 (4)	0.023 (2)	−0.012 (2)	0.0088 (19)	−0.012 (2)
C17	0.038 (2)	0.036 (3)	0.031 (2)	0.004 (2)	0.0090 (19)	−0.006 (2)
C11	0.033 (2)	0.023 (3)	0.039 (3)	−0.0059 (19)	0.0151 (19)	−0.0070 (19)
N6	0.029 (2)	0.054 (3)	0.042 (2)	0.0132 (19)	0.0003 (17)	−0.006 (2)
C20	0.035 (2)	0.035 (3)	0.027 (2)	−0.002 (2)	0.0065 (18)	0.004 (2)
C9	0.0205 (19)	0.024 (2)	0.021 (2)	0.0015 (17)	−0.0008 (15)	0.0011 (17)
N2	0.046 (2)	0.044 (3)	0.037 (2)	−0.0142 (19)	0.0242 (18)	−0.0041 (19)
C14	0.047 (3)	0.058 (4)	0.033 (3)	0.007 (2)	0.014 (2)	0.014 (2)
C19	0.039 (3)	0.041 (3)	0.030 (3)	0.009 (2)	−0.0005 (19)	−0.016 (2)
C22	0.043 (3)	0.065 (4)	0.026 (2)	−0.006 (3)	0.005 (2)	0.023 (2)
C21	0.034 (3)	0.081 (4)	0.036 (3)	0.001 (3)	0.002 (2)	0.031 (3)
C18	0.036 (3)	0.057 (4)	0.042 (3)	0.005 (2)	−0.004 (2)	−0.023 (3)
N4	0.055 (3)	0.064 (3)	0.058 (3)	0.011 (2)	0.034 (2)	−0.005 (2)
C16	0.037 (3)	0.051 (3)	0.049 (3)	0.008 (2)	0.022 (2)	0.006 (3)
C15	0.064 (4)	0.084 (5)	0.054 (4)	0.000 (3)	0.036 (3)	0.000 (3)
C12	0.059 (3)	0.069 (4)	0.036 (3)	−0.011 (3)	0.026 (2)	−0.008 (3)
O11	0.042 (2)	0.055 (3)	0.082 (3)	0.0004 (18)	0.0066 (19)	0.027 (2)
N9	0.042 (2)	0.043 (3)	0.076 (3)	0.006 (2)	0.014 (2)	−0.014 (2)
C24	0.083 (4)	0.071 (5)	0.078 (5)	−0.021 (4)	0.044 (4)	−0.027 (4)
C23	0.068 (4)	0.048 (4)	0.062 (4)	−0.014 (3)	0.008 (3)	−0.002 (3)
C25	0.091 (6)	0.083 (6)	0.173 (10)	0.048 (5)	−0.014 (6)	−0.025 (6)

### Geometric parameters (Å, º)

**Table:**

Cu1—N1	2.003 (4)	C3—C5	1.389 (5)
Cu1—N3	2.025 (4)	C6—C5	1.393 (5)
Cu1—O1	1.984 (3)	C6—C8	1.398 (5)
Cu1—O5^i^	1.985 (3)	C8—C9	1.506 (5)
Cu1—O9	2.344 (3)	C5—H5	0.9300
Cu2—N5	1.985 (3)	C13—C12	1.335 (7)
Cu2—N7	1.980 (3)	C13—H13	0.9300
Cu2—O3	2.243 (3)	C17—N6	1.327 (6)
Cu2—O7^ii^	2.003 (3)	C17—H17	0.9300
Cu2—O10	2.043 (3)	C11—N2	1.337 (6)
O3—C4	1.257 (5)	C11—H11	0.9300
O4—C4	1.245 (5)	N6—C18	1.358 (6)
O2—C1	1.240 (5)	N6—H6N	0.8600
O1—C1	1.282 (5)	C20—H20	0.9300
O5—C7	1.256 (5)	N2—C12	1.340 (6)
O5—Cu1^iii^	1.985 (3)	N2—H2N	0.8600
O6—C7	1.247 (5)	C14—C15	1.339 (8)
O8—C9	1.228 (5)	C14—H14	0.9300
O7—C9	1.273 (5)	C19—C18	1.344 (6)
O7—Cu2^iv^	2.003 (3)	C19—H19	0.9300
O10—H10A	0.8500	C22—C21	1.344 (7)
O10—H10B	0.8500	C22—H22	0.9300
O9—H9B	0.8499	C21—H21	0.9300
O9—H9C	0.8500	C18—H18	0.9300
N1—C11	1.312 (5)	N4—C16	1.335 (7)
N1—C13	1.367 (6)	N4—C15	1.335 (7)
N7—C20	1.323 (6)	N4—H4N	0.8600
N7—C22	1.380 (5)	C16—H16	0.9300
N3—C16	1.327 (6)	C15—H15	0.9300
N3—C14	1.348 (6)	C12—H12	0.9300
N5—C17	1.325 (6)	O11—C23	1.217 (7)
N5—C19	1.376 (5)	N9—C23	1.313 (8)
C7—C6	1.512 (5)	N9—C24	1.429 (8)
C2—C10	1.378 (5)	N9—C25	1.487 (8)
C2—C3	1.406 (5)	C24—H24A	0.9600
C2—C1	1.503 (5)	C24—H24B	0.9600
C4—C3	1.514 (5)	C24—H24C	0.9600
C10—C8	1.393 (5)	C23—H23	0.9300
C10—H10	0.9300	C25—H25A	0.9600
N8—C20	1.327 (6)	C25—H25B	0.9600
N8—C21	1.342 (6)	C25—H25C	0.9600
N8—H8N	0.8600		
			
O1—Cu1—O5^i^	176.77 (12)	C3—C5—C6	120.8 (4)
O1—Cu1—N1	88.65 (13)	C3—C5—H5	119.6
O5^i^—Cu1—N1	89.92 (13)	C6—C5—H5	119.6
O1—Cu1—N3	90.46 (13)	C12—C13—N1	109.2 (4)
O5^i^—Cu1—N3	90.31 (13)	C12—C13—H13	125.4
N1—Cu1—N3	167.36 (15)	N1—C13—H13	125.4
O1—Cu1—O9	98.32 (12)	N5—C17—N6	111.0 (4)
O5^i^—Cu1—O9	84.71 (12)	N5—C17—H17	124.5
N1—Cu1—O9	96.07 (13)	N6—C17—H17	124.5
N3—Cu1—O9	96.53 (14)	N1—C11—N2	111.8 (4)
N7—Cu2—N5	172.01 (15)	N1—C11—H11	124.1
N7—Cu2—O7^ii^	94.38 (13)	N2—C11—H11	124.1
N5—Cu2—O7^ii^	88.09 (13)	C17—N6—C18	107.6 (4)
N7—Cu2—O10	87.98 (13)	C17—N6—H6N	126.2
N5—Cu2—O10	88.73 (13)	C18—N6—H6N	126.2
O7^ii^—Cu2—O10	173.16 (12)	N7—C20—N8	111.2 (4)
N7—Cu2—O3	91.92 (13)	N7—C20—H20	124.4
N5—Cu2—O3	95.39 (13)	N8—C20—H20	124.4
O7^ii^—Cu2—O3	95.99 (11)	O8—C9—O7	124.6 (4)
O10—Cu2—O3	90.34 (12)	O8—C9—C8	120.6 (4)
C4—O3—Cu2	125.3 (3)	O7—C9—C8	114.6 (4)
C1—O1—Cu1	106.6 (2)	C11—N2—C12	106.3 (4)
C7—O5—Cu1^iii^	113.6 (2)	C11—N2—H2N	126.8
C9—O7—Cu2^iv^	114.2 (3)	C12—N2—H2N	126.8
Cu2—O10—H10A	109.2	C15—C14—N3	110.8 (5)
Cu2—O10—H10B	109.3	C15—C14—H14	124.6
H10A—O10—H10B	109.5	N3—C14—H14	124.6
Cu1—O9—H9B	109.2	C18—C19—N5	108.8 (4)
Cu1—O9—H9C	109.3	C18—C19—H19	125.6
H9B—O9—H9C	109.5	N5—C19—H19	125.6
C11—N1—C13	104.8 (4)	C21—C22—N7	109.6 (4)
C11—N1—Cu1	124.3 (3)	C21—C22—H22	125.2
C13—N1—Cu1	129.3 (3)	N7—C22—H22	125.2
C20—N7—C22	104.5 (4)	N8—C21—C22	106.4 (4)
C20—N7—Cu2	128.8 (3)	N8—C21—H21	126.8
C22—N7—Cu2	126.5 (3)	C22—C21—H21	126.8
C16—N3—C14	103.6 (4)	C19—C18—N6	107.0 (4)
C16—N3—Cu1	124.9 (3)	C19—C18—H18	126.5
C14—N3—Cu1	129.4 (3)	N6—C18—H18	126.5
C17—N5—C19	105.7 (4)	C16—N4—C15	106.2 (5)
C17—N5—Cu2	126.0 (3)	C16—N4—H4N	126.9
C19—N5—Cu2	128.3 (3)	C15—N4—H4N	126.9
O6—C7—O5	125.6 (3)	N3—C16—N4	112.3 (5)
O6—C7—C6	117.8 (4)	N3—C16—H16	123.9
O5—C7—C6	116.7 (3)	N4—C16—H16	123.9
C10—C2—C3	119.5 (3)	N4—C15—C14	107.1 (5)
C10—C2—C1	121.4 (4)	N4—C15—H15	126.4
C3—C2—C1	119.0 (3)	C14—C15—H15	126.4
O4—C4—O3	127.1 (4)	C13—C12—N2	107.8 (5)
O4—C4—C3	119.1 (4)	C13—C12—H12	126.1
O3—C4—C3	113.6 (3)	N2—C12—H12	126.1
O2—C1—O1	124.0 (3)	C23—N9—C24	123.4 (5)
O2—C1—C2	119.0 (4)	C23—N9—C25	120.7 (7)
O1—C1—C2	116.9 (3)	C24—N9—C25	115.0 (6)
C2—C10—C8	121.6 (4)	N9—C24—H24A	109.5
C2—C10—H10	119.2	N9—C24—H24B	109.5
C8—C10—H10	119.2	H24A—C24—H24B	109.5
C20—N8—C21	108.2 (4)	N9—C24—H24C	109.5
C20—N8—H8N	125.9	H24A—C24—H24C	109.5
C21—N8—H8N	125.9	H24B—C24—H24C	109.5
C5—C3—C2	119.3 (3)	O11—C23—N9	125.4 (6)
C5—C3—C4	118.6 (3)	O11—C23—H23	117.3
C2—C3—C4	121.9 (3)	N9—C23—H23	117.3
C5—C6—C8	119.9 (3)	N9—C25—H25A	109.5
C5—C6—C7	119.6 (3)	N9—C25—H25B	109.5
C8—C6—C7	120.4 (3)	H25A—C25—H25B	109.5
C10—C8—C6	118.8 (3)	N9—C25—H25C	109.5
C10—C8—C9	119.3 (3)	H25A—C25—H25C	109.5
C6—C8—C9	121.7 (3)	H25B—C25—H25C	109.5
			
N7—Cu2—O3—C4	98.4 (3)	C1—C2—C3—C4	−7.1 (5)
N5—Cu2—O3—C4	−78.3 (3)	O4—C4—C3—C5	−78.2 (5)
O7^ii^—Cu2—O3—C4	−167.0 (3)	O3—C4—C3—C5	98.4 (4)
O10—Cu2—O3—C4	10.4 (3)	O4—C4—C3—C2	107.7 (4)
O5^i^—Cu1—O1—C1	−13 (2)	O3—C4—C3—C2	−75.7 (5)
N1—Cu1—O1—C1	−76.3 (3)	O6—C7—C6—C5	−141.6 (4)
N3—Cu1—O1—C1	91.1 (3)	O5—C7—C6—C5	38.7 (5)
O9—Cu1—O1—C1	−172.2 (3)	O6—C7—C6—C8	33.9 (5)
O1—Cu1—N1—C11	142.5 (4)	O5—C7—C6—C8	−145.8 (4)
O5^i^—Cu1—N1—C11	−34.6 (3)	C2—C10—C8—C6	−3.0 (6)
N3—Cu1—N1—C11	56.4 (7)	C2—C10—C8—C9	172.2 (4)
O9—Cu1—N1—C11	−119.3 (3)	C5—C6—C8—C10	2.0 (6)
O1—Cu1—N1—C13	−20.6 (4)	C7—C6—C8—C10	−173.5 (3)
O5^i^—Cu1—N1—C13	162.3 (4)	C5—C6—C8—C9	−173.0 (4)
N3—Cu1—N1—C13	−106.6 (7)	C7—C6—C8—C9	11.5 (6)
O9—Cu1—N1—C13	77.7 (4)	C2—C3—C5—C6	−1.3 (6)
N5—Cu2—N7—C20	−105.7 (10)	C4—C3—C5—C6	−175.5 (4)
O7^ii^—Cu2—N7—C20	2.1 (4)	C8—C6—C5—C3	0.1 (6)
O10—Cu2—N7—C20	−171.5 (4)	C7—C6—C5—C3	175.6 (3)
O3—Cu2—N7—C20	98.3 (4)	C11—N1—C13—C12	−0.1 (6)
N5—Cu2—N7—C22	79.8 (11)	Cu1—N1—C13—C12	165.4 (4)
O7^ii^—Cu2—N7—C22	−172.4 (4)	C19—N5—C17—N6	−0.8 (5)
O10—Cu2—N7—C22	14.0 (4)	Cu2—N5—C17—N6	−178.3 (3)
O3—Cu2—N7—C22	−76.2 (4)	C13—N1—C11—N2	0.2 (5)
O1—Cu1—N3—C16	−39.4 (4)	Cu1—N1—C11—N2	−166.3 (3)
O5^i^—Cu1—N3—C16	137.5 (4)	N5—C17—N6—C18	0.2 (6)
N1—Cu1—N3—C16	46.5 (8)	C22—N7—C20—N8	0.7 (5)
O9—Cu1—N3—C16	−137.8 (4)	Cu2—N7—C20—N8	−174.8 (3)
O1—Cu1—N3—C14	160.2 (4)	C21—N8—C20—N7	−0.7 (6)
O5^i^—Cu1—N3—C14	−23.0 (4)	Cu2^iv^—O7—C9—O8	−14.0 (5)
N1—Cu1—N3—C14	−114.0 (6)	Cu2^iv^—O7—C9—C8	160.5 (2)
O9—Cu1—N3—C14	61.7 (4)	C10—C8—C9—O8	58.3 (5)
N7—Cu2—N5—C17	−156.0 (9)	C6—C8—C9—O8	−126.6 (4)
O7^ii^—Cu2—N5—C17	95.8 (4)	C10—C8—C9—O7	−116.4 (4)
O10—Cu2—N5—C17	−90.3 (4)	C6—C8—C9—O7	58.6 (5)
O3—Cu2—N5—C17	−0.1 (4)	N1—C11—N2—C12	−0.2 (6)
N7—Cu2—N5—C19	27.0 (12)	C16—N3—C14—C15	0.3 (6)
O7^ii^—Cu2—N5—C19	−81.2 (4)	Cu1—N3—C14—C15	163.9 (4)
O10—Cu2—N5—C19	92.8 (4)	C17—N5—C19—C18	1.1 (6)
O3—Cu2—N5—C19	−177.0 (4)	Cu2—N5—C19—C18	178.6 (4)
Cu1^iii^—O5—C7—O6	3.3 (5)	C20—N7—C22—C21	−0.4 (6)
Cu1^iii^—O5—C7—C6	−177.1 (2)	Cu2—N7—C22—C21	175.2 (4)
Cu2—O3—C4—O4	13.8 (5)	C20—N8—C21—C22	0.4 (7)
Cu2—O3—C4—C3	−162.4 (2)	N7—C22—C21—N8	0.0 (7)
Cu1—O1—C1—O2	−6.3 (5)	N5—C19—C18—N6	−1.1 (7)
Cu1—O1—C1—C2	175.1 (3)	C17—N6—C18—C19	0.5 (6)
C10—C2—C1—O2	152.4 (4)	C14—N3—C16—N4	0.2 (6)
C3—C2—C1—O2	−26.0 (6)	Cu1—N3—C16—N4	−164.4 (3)
C10—C2—C1—O1	−28.9 (6)	C15—N4—C16—N3	−0.6 (7)
C3—C2—C1—O1	152.7 (4)	C16—N4—C15—C14	0.8 (7)
C3—C2—C10—C8	1.8 (6)	N3—C14—C15—N4	−0.7 (7)
C1—C2—C10—C8	−176.7 (4)	N1—C13—C12—N2	0.0 (7)
C10—C2—C3—C5	0.3 (6)	C11—N2—C12—C13	0.1 (6)
C1—C2—C3—C5	178.8 (3)	C24—N9—C23—O11	−5.1 (10)
C10—C2—C3—C4	174.4 (4)	C25—N9—C23—O11	−173.7 (6)

Symmetry codes: (i) *x*+1, −*y*+3/2, *z*+1/2; (ii) *x*, −*y*+3/2, *z*+1/2; (iii) *x*−1, −*y*+3/2, *z*−1/2; (iv) *x*, −*y*+3/2, *z*−1/2.

### Hydrogen-bond geometry (Å, º)

**Table:**

*D*—H···*A*	*D*—H	H···*A*	*D*···*A*	*D*—H···*A*
N2—H2*N*···O3^v^	0.86	2.01	2.807 (5)	153
N4—H4*N*···O7^ii^	0.86	2.35	3.099 (5)	146
N6—H6*N*···O6^i^	0.86	2.03	2.733 (5)	138
N8—H8*N*···O2^vi^	0.86	2.35	2.952 (5)	127
N8—H8*N*···O4^vi^	0.86	2.26	3.033 (6)	149
O9—H9*B*···O11^vii^	0.85	2.06	2.909 (5)	177
O9—H9*C*···O11^vi^	0.85	2.02	2.805 (6)	153
O10—H10*A*···O8^viii^	0.85	1.90	2.655 (4)	147
O10—H10*B*···O4	0.85	2.24	2.703 (5)	114

Symmetry codes: (i) *x*+1, −*y*+3/2, *z*+1/2; (ii) *x*, −*y*+3/2, *z*+1/2; (v) *x*+1, *y*, *z*; (vi) *x*−1, *y*, *z*; (vii) −*x*+2, −*y*+1, −*z*+1; (viii) −*x*, *y*+1/2, −*z*+1/2.
